# Relationship of shear wave elastography anisotropy with tumor stem cells and epithelial-mesenchymal transition in breast cancer

**DOI:** 10.1186/s12880-021-00707-z

**Published:** 2021-11-17

**Authors:** Xiaoling Leng, Rexida Japaer, Haijian Zhang, Mila Yeerlan, Fucheng Ma, Jianbing Ding

**Affiliations:** 1 Department of Ultrasound, Affiliated Tumor Hospital of Xinjiang Medical University, No. 789 Suzhou East Road, Xinshi District, Urumqi, 830011 People’s Republic of China; 2 School of Basic Medicine, Xinjiang Medical University, No. 567 Shangde North Road, Urumqi, 830017 Xinjiang People’s Republic of China

**Keywords:** Anisotropy, Breast cancer, Cancer stem cells, Epithelial-mesenchymal transition, Shear wave elastography (SWE)

## Abstract

**Background:**

This study is to examine the feasibility of shear wave elastography (SWE) anisotropy in assessing the prognosis of breast cancer.

**Methods:**

We enrolled 119 breast cancer patients from January 2017 to October 2019. SWE was performed before operation. Emax (maximum elasticity value), Emean (average elasticity value), Esd (standard deviation of the lesion elasticity value), Eratio (elasticity value of adipose tissue), anisotropy coefficient and difference were recorded. After operation, we collected clinical pathological data, and performed immunohistochemistry and real-time PCR tests on CD44, CD24, E-cadherin, β-catenin, vimentin and N-cadherin. Finally, we analyzed the correlation among parameters of SWE, anisotropy and clinicopathology, and markers of CSCs (cancer stem cells) and EMT (epithelial-mesenchymal transition).

**Results:**

Emax, Emean and Esd of the cross section were higher than those of the longitudinal section. Breast cancer with a higher elastic modulus was often accompanied by a hyperechoic halo, which was manifested as mixed echo and post-echo attenuation, and was accompanied by a higher BI-RADS (breast imaging reporting and data system) classification. When breast cancer had hyperechoic halo and weakened posterior echo, SWE of the lesion showed more obvious anisotropy. In addition, larger diameter of the longitudinal section indicated higher stiffness of the cross section. Correlation analysis showed that E-cadherin was negatively correlated with SWE in longitudinal section. CD44, N-cadherin, β-catenin were positively correlated with SWE in longitudinal and cross sections. Vimentin and CD24 had no correlation with SWE parameters.

**Conclusion:**

SWE of breast cancer is anisotropic. The cross-sectional SWE is better than the longitudinal SWE, Emax is better than Emean, the anisotropy of SWE is better than SWE, and the anisotropy factor is better than the anisotropy difference.

## Background

Studies [1, 2] have shown that tumor stiffness is determined by the stiffness of the matrix. The extracellular matrix is composed of a network of biopolymerized fibers. The density of extracellular matrix is determined by the collagen content, fiber thickness, internal fiber cross-linking degree, and the porosity of the extracellular matrix, which further determine the mechanical properties and rheological properties of the extracellular matrix [3]. With the abnormal deposition and cross-linking of the extracellular matrix, as well as the increase in matrix stiffness, the mechanical properties of the tumor microenvironment will change [4], causing the stiffness of tumor tissue [5]. Tumor stiffness can be reflected on ultrasonic images. However, ultrasonic images are affected by uncertainties and/or inaccuracies of various kinds which, among other things, determine an extremely low quality grayscale image contrast. This contrast has been treated by using fuzzy techniques precisely [6, 7]. Among them, shear wave elastography (SWE) is a stable ultrasonic elastic technology with high repeatability and is independent of external pressure, which reflects the stiffness of the tissue to a certain extent [8]. By exciting and precisely controlling the propagation and capture of shear waves in the human body, SWE presents the elastic modulus of the tissue in real time. However, breast cancer is a highly heterogeneous tumor. In clinical practice, we observe that SWE of breast cancer is anisotropic. SWE quantitative parameters can quantify anisotropy and have certain research value. With the occurrence and development of breast cancer, its physical and chemical properties will change [9]. It is found that the anisotropy of breast lesions may be related to the degree of tumor malignancy [10], which is only limited to diagnostic evaluation. In clinic, a method to non-invasively assess the prognosis of breast cancer before surgery is needed.

Although the clinicopathological features of tumor size, pathological type, axillary lymph node involvement and molecular subtypes of breast cancer are all related to the prognosis, studies have shown that cancer stem cells (CSCs) and epithelial-mesenchymal transition (EMT) can better reflect the prognosis of breast cancer than clinicopathological features [11], and they are the key regulators of breast cancer aggressiveness [12]. CSCs of breast cancer have self-renewal and multidirectional differentiation capabilities, which are closely related to tumor occurrence, proliferation, metastasis, and drug resistance [12]. EMT causes epithelial cells to lose tight junctions and polarity, and acquire the characteristics of mesenchymal cells, which is always accompanied by up-regulation of N-cadherin and down-regulation of E-cadherin, and is closely related to tumor invasion, metastasis and treatment resistance [13].

One previous study has quantitatively assessed the relationship between breast cancer ultrasound characteristics and biological characteristics through automated imaging omics methods, which showed that the accuracy of ultrasound in predicting breast cancer hormone receptor expression level was 67.7% [14]. This indicates that tumor features at the genetic and cellular level could be reflected through ultrasound imaging. In this study, through correlation analysis, we aim to use SWE to predict the prognostic characteristics of breast cancer at the cellular and molecular levels. We analyzed the anisotropic quantitative parameters of SWE reflecting tumor stiffness and analyzed their correlation with prognostic factors. We measured the expression of CSCs and EMT markers in breast cancer tissues, as well as their correlations with SWE parameters. Our findings may guide the assessment the clinical prognosis of breast using a non-invasive and convenient imaging method.

## Methods

### Subjects

We included patients who underwent breast cancer surgery at the Department of Breast Surgery of Xinjiang Cancer Hospital from January 2017 to October 2018 and were confirmed to have breast cancer by pathological examination after the operation. All patients underwent routine ultrasound and SWE examinations before surgery, and clinical pathological data were collected after surgery. The exclusion criteria were: (1) patients had undergone neoadjuvant chemotherapy or radiotherapy; (2) patients had recently received a needle biopsy; (3) patients had a history of prosthesis implantation during pregnancy or lactation; (4) patients had a history of surgery on the ipsilateral breast, and had scar tissue adjacent to the lesion; (5) patients with non-mass lesions (such as simple diffuse calcification) on ultrasound; (6) patients with incomplete histopathological data; (7) patients with lesions that cannot be covered by the SWE sampling frame. Finally, 119 patients with breast cancer were included as the study cohort. The age range of the patients was 25–84 years old, with an average age of 48.75 ± 12.12 years old. Tumor samples were obtained by puncture.

Written informed consent was obtained from every patient and the study was approved by the ethics review board of the Affiliated Tumor Hospital of Xinjiang Medical University (No.: G-202102).

### Conventional ultrasound and SWE

SuperSonic Imagine’s Aixplorer (SuperSonic Imagine, Aix-en-Provence, France), which had built-in real-time SWE, and was with L1 5–4 linear array probe and frequency 4–15 MHz, was used. After the patients’ bilateral breast and axilla were fully exposed, we performed continuous radial scans centered on the nipple to observe in detail the tumor size, shape, internal echo, boundaries, borders, intralesional calcification, posterior echo attenuation, and blood flow. First, we acquired the best conventional ultrasound image, and then switched to SWE mode (default range 0–180 kPa, "STD" mode (standard mode)). When the lesion was larger or the site was deeper, we adjusted the ultrasound to the "PEN" mode (penetration mode) to perform qualitative and quantitative SWE observation of the lesion. Subsequently, the probe was rotated along the center of the lesion to the vertical section (longitudinal section) of the largest diameter section, and the previous operation was repeated. Finally, we accessed three independent SWE images in the horizontal and vertical sections, respectively, with the built-in "Q-Box" quantitative software. The sampling frame included the entire lesion and the adjacent hard areas as much as possible. The maximum elasticity value (Emax), the average elasticity value (Emean) and the standard deviation of the lesion elasticity value (Esd) of the entire lesion were recorded. The ratio of the average elasticity value of the hardest part of the lesion to the elasticity value of adipose tissue (Eratio) was calculated. The anisotropy coefficient for each quantitative parameter, including anisotropic difference (AD) and anisotropy factor (AF), is calculated as follows: AD = (cross-section elasticity value) − (longitudinal-section elasticity value); AF = [(cross-section elasticity value) − (longitudinal-section elasticity value)]^2^.

### Immunohistochemistry

The tumor tissues were sliced and immunohistochemically stained by Envision two-step method to detect EMT markers, including E-cadherin, β-catenin, vimentin, N-cadherin, and CSCs markers CD44 and CD24. Briefly, after antigen retrieval, the endogenous peroxidase was inactivated with 3% H_2_O_2_. Then, the primary antibodies of anti-E-Cadherin (ab40772, Abcam), anti-β-catenin (ab32572, Abcam), anti-Vimenti (ab8978, Abcam), anti-N-cadherin (ab76011, Abcam), anti-CD44 (ab157107, Abcam), and anti-Human CD24 (12-0247-42, Thermo) were added and incubated at 4 °C overnight. On the second day, the sample was incubated with secondary antibody for 1 h at room temperature. After that, the samples were washed 3 times with PBS and developed for 20 min at room temperature. Five fields were randomly observed, and 100 tumor cells were counted. The staining intensity and the percentage of tumor cells with positive staining were used for scoring. Among them, the staining intensity was divided into four levels: negative (no staining), weakly positive (light yellow), positive (brown yellow), and strongly positive (tan) staining. In addition, the percentage of positive tumor cells was also divided into four grades: < 5% (0 points), 5–25% (1 points), 25–50% (2 points), 50–75% (3 points), and > 75% (4 points). Finally, according to the score, the staining results were divided into negative (−), very weak positive (±), weak positive (+), positive (+++), and strong positive (+++) staining.

### Real-time PCR

Total RNAs were extracted from breast cancer tissues with TRLZOL (Transgene, ET111) and reverse transcribed into cDNA with TransScript One-Step gDNA Removal and cDNA Synthesis SuperMix (Transgene, AT311). The real-time PCR was performed with QuantiNava SYBR Green Kit (208,054, Qiagen, USA) and on ABI7500 (ABI, USA). The primer sequences for E-cadherin, β-catenin, vimentin, N-cadherin, CD44, CD24 and *actin* were shown in Table 1. The reaction system was: 2 × SYBR Green Select Mix (5 μl), Forward Primer (0.7 μl), Reverse Primer (0.7 μl), ROX (0.05 μl), cDNA (1 μl), and RNase-free Water (Up to 10 μl). The reaction conditions were pre-denaturation at 95 °C for 2 min; denaturation at 95 °C for 30 s, annealing/extension at 60 °C for 30 s, 40 cycles. The 2^−ΔΔCt^ method was used to calculate the relative expression of each gene.

**Table 1:** Real-time PCR primer sequences

Genes	Primers	Sequence (5' to 3')	Product (bp)
E-cadherin	Forward	CGAGAGCTACACGTTCACGG	119
	Reverse	GGGTGTCGAGGGAAAAATAGG	
β-catenin	Forward	AGCTTCCAGACACGCTATCAT	98
	Reverse	CGGTACAACGAGCTGTTTCTAC	
Vimentin	Forward	AGTCCACTGAGTACCGGAGAC	98
	Reverse	CATTTCACGCATCTGGCGTTC	
N-cadherin	Forward	AGCCAACCTTAACTGAGGAGT	136
	Reverse	GGCAAGTTGATTGGAGGGATG	
CD44	Forward	CTGCCGCTTTGCAGGTGTA	109
	Reverse	CATTGTGGGCAAGGTGCTATT	
CD24	Forward	CTCCTACCCACGCAGATTTATTC	166
	Reverse	AGAGTGAGACCACGAAGAGAC	
hsa actin	Forward	ACAGAGCCTCGCCTTTGCC	250
	Reverse	GAGGATGCCTCTCTTGCTCTG	

### Statistical analysis

Data analysis was performed using SPSS21.0 software, and *P* < 0.05 was considered as statistically significant. All data are expressed as mean ± standard deviation (SD). If the data conforms to the normal distribution, one-way analysis of variance was used for multiple comparisons followed by the Sidak method (uniform variance) or the Tamhane method (non-uniform variance). If the data do not conform to the normal distribution, logarithmic transformation of the data was first performed to normalize the data, and then one-way analysis of variance or the Wilcoxon rank sum test was used. Pearson’s correlation analysis was used for correlation analysis.

## Results

### The SWE of breast cancer is anisotropic

**Fig. 1:**
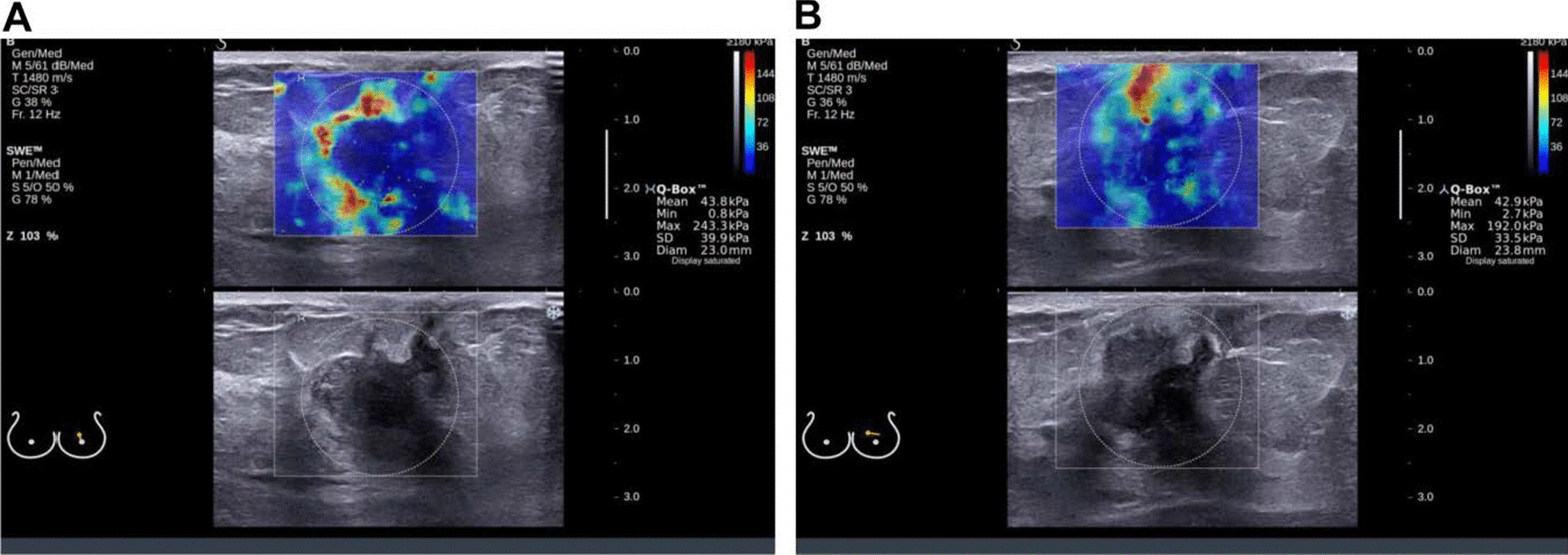
The anisotropy of SWE of breast cancer on cross section and longitudinal section on ultrasound. The representative ultrasound images of a patient of 66 years old with triple-negative breast cancer were shown. The size of the lesion was about 1.9 × 1.1 cm, with histological grade 3 and clinical stage 1. The cross section of SWE had a higher elastic modulus value than the longitudinal section, showing anisotropy. A: The elastic modulus parameters Emax, Emean, Esd, Eratio of breast cancer on cross section of breast cancer SWE. B: The elastic modulus parameters Emax, Emean, Esd, Eratio of on the longitudinal section of breast cancer SWE

In order to clarify whether there is anisotropy in SWE of breast cancer, we first explored the difference of SWE parameters in longitudinal and cross sections. The results showed that the Emax, Emean and Esd of all the cross-section lesions were significantly higher than the longitudinal section (*P* < 0.05), in which Emax: (139.87 ± 92.64) kPa vs. (133.28 ± 90.80) kPa, *P* = 0.001; Emean: (45.22 ± 26.54) kPa vs. (42.65 ± 24.92) kPa, *P* = 0.001; Esd: (15.971 ± 9.096) kPa vs. (18.806 ± 14.482) kPa, *P* = 0.0161. However, there was no statistically significant difference in Eratio between the cross section and the longitudinal Sect. (11.40 ± 7.79 vs. 10.96 ± 7.50, *P* = 0.075). As shown in Fig. 1, the breast cancer lesion had anisotropy of SWE parameters, and the SWE elastic modulus in the cross section was significantly higher than that in the longitudinal section. The above results indicate that breast cancer SWE is anisotropic, and the elastic modulus value of the cross section is higher than that of the longitudinal section.

### The SWE and anisotropy parameters of breast cancer have a strong relation with conventional ultrasound signs

In order to clarify whether breast cancers with different ultrasound characteristics have different SWE anisotropies, we then analyzed the differences between SWE parameters, conventional ultrasound features and BI-RADS (breast imaging reporting and data system) classification (Table 2). The results showed that SWE parameters were significantly related with breast cancer boundary, internal echo, posterior echo and BI-RADS. This indicates that breast cancer with higher elastic modulus tends to be accompanied by hyperechoic halo, showing mixed echo and posterior echo attenuation, and has a higher BI-RADS classification. Among them, the relation of Emax and Emean with conventional ultrasound signs was stronger than that of Esd and Eratio. The elastic modulus of the cross and longitudinal sections of breast cancer had obvious relation with the boundary of the lesion, internal echo, and posterior echo. However, the relation between the elastic modulus of the cross section and the BI-RADS classification was better than that of the longitudinal section. In addition, the anisotropy parameters of breast cancer were significantly related to conventional ultrasound signs. When there were hyperechoic halo and attenuated posterior echoes in breast cancer lesion, the SWE of the lesions presented more obvious anisotropy, the blood supply of the lesion was less, and the anisotropy of SWE was more obvious. However, the anisotropy of SWE was not related to internal echo and BI-RADS classification. In addition, the anisotropy parameters of Admax, Admean, Adsd, Afratio were positively correlated with the distance between the mass and skin (Table 3). However, the Young's modulus values of SWE were not related to the distance of the mass surface and body surface (Table 4). In addition, Admax, Admean, ADratio, Afmax, Afmean had a significant positive correlation with the maximum diameters of the ultrasonic cross section and longitudinal section. Each SWE parameter of the cross and longitudinal sections was positively correlated with their respective maximum diameter. However, the SWE parameters of the cross section were also positively correlated with the largest diameter of the longitudinal section (Table 4). These results indicate that the larger the lesion, the harder the breast cancer and the more obvious the anisotropy. Moreover, the larger the diameter of the longitudinal section, the higher the stiffness of the cross section.

**Table 2:** Differences in SWE parameters of cross section and longitudinal section of breast cancer of conventional ultrasound

Items	Grouping	Cases	E-maximum cross section diameter(mm)	E-maximum longitudinal section diameter(mm)	Emax (cross section) (k Pa)	Emax (longitudinal section) (k Pa)	Emean (cross section) (k Pa)	Emean (longitudinal section) (k Pa)	Esd (cross section) (k Pa)	Esd (longitudinal section) (k Pa)	Eratio (cross section)	Eratio (longitudinal section)
Boundary	Closely connected	62	17.912 ± 4.994	17.321 ± 4.354	86.060 ± 45.765	82.914 ± 41.734	55.481 ± 34.786	60.905 ± 31.074	13.460 ± 5.205	15.595 ± 6.374	7.350 ± 4.199	7.669 ± 3.168
	Hyperechoic halo	57	20.514 ± 6.944	19.681 ± 6.713	120.400 ± 68.395*	117.086 ± 75.431*	79.765 ± 43.264**	79.949 ± 48.472	18.822 ± 11.519	22.451 ± 19.556	10.386 ± 7.429	8.578 ± 6.872
Internal echo	Mixed echo	38	21.589 ± 5.632	19.822 ± 4.896	142.328 ± 63.540	155.900 ± 82.714	90.800 ± 42.617	102.994 ± 55.940	21.889 ± 10.111	31.072 ± 24.808	10.678 ± 8.543	10.406 ± 8.174
	Low echo	81	18.405 ± 6.071	18.015 ± 5.854	90.285 ± 53.415**	82.105 ± 42.272**	59.789 ± 37.496**	60.036 ± 29.569**	14.225 ± 8.061**	15.187 ± 6.310**	8.210 ± 5.109	7.413 ± 3.802
Posterior echo	No change	47	17.444 ± 6.006	16.759 ± 6.151	92.926 ± 57.482	96.735 ± 70.160	59.650 ± 36.002	69.303 ± 43.949	13.921 ± 8.747	19.674 ± 19.992	8.535 ± 7.042	8.332 ± 6.724
	Enhance	35	17.455 ± 5.575	17.214 ± 4.701	71.495 ± 42.604	67.145 ± 23.514	44.218 ± 28.780	49.309 ± 21.402	11.682 ± 3.602	13.023 ± 2.364	6.427 ± 5.031	6.864 ± 3.586
	Attenuation	37	23.226 ± 4.788**	22.052 ± 4.085**	145.083 ± 54.085**	132.539 ± 59.082**	99.156 ± 37.776**	90.217 ± 42.165**	23.104 ± 9.385**	23.056 ± 9.234**	11.365 ± 4.500**	8.922 ± 3.782
Blood flow	None or undetectable	21	14.425 ± 1.521	14.813 ± 1.968	132.263 ± 56.249	129.200 ± 49.720	79.550 ± 25.219	81.863 ± 26.564	17.100 ± 5.284	18.513 ± 5.406	9.275 ± 3.176	9.463 ± 3.689
	Intra-lesion signal	74	19.292 ± 6.277	18.720 ± 5.822	98.741 ± 57.638	93.220 ± 59.850	65.339 ± 39.775	67.433 ± 39.759	15.551 ± 9.197	18.552 ± 15.679	9.369 ± 6.597	8.287 ± 5.663
	Increased diffuse blood flow around the lesion	24	21.910 ± 5.323*	19.530 ± 6.023	98.800 ± 72.969	109.460 ± 79.270	65.940 ± 55.564	74.780 ± 57.509	17.630 ± 11.226	20.590 ± 12.326	4.730 ± 1.572*	5.830 ± 2.108
BI-RADS	4a	18	14.220 ± 6.737	12.380 ± 2.848	87.480 ± 79.255	71.800 ± 10.133	50.520 ± 45.899	52.520 ± 30.195	10.840 ± 8.271	11.700 ± 0.822	5.920 ± 4.272	4.440 ± 0.329
	4b	22	13.025 ± 5.662	14.988 ± 5.192	51.588 ± 10.885	69.063 ± 15.450	26.788 ± 4.175	44.238 ± 21.487	8.725 ± 1.914	13.863 ± 0.650	2.800 ± 0.949	8.163 ± 3.551
	4c, 5	79	20.242 ± 5.526**	19.302 ± 5.491**	109.382 ± 59.035*	104.592 ± 66.123	72.948 ± 39.889**	69.339 ± 39.770	17.238 ± 9.196**	19.944 ± 15.601*	9.712 ± 6.111**	8.364 ± 5.502*

Emax = maximum elasticity; Emean = mean elasticity; Esd = stand deviation; Eratio = elasticity ratio; BI-RADS = breast imaging reporting and data system. Compared with corresponding subgroup, *P < 0.05, **P < 0.01

**Table 3:** Differences of breast cancer SWE anisotropy parameters in conventional ultrasound

Items	Grouping	N	Afmax	Afmean	Afsd	Afratio	Admax (k Pa)	Admean (k Pa)	Adsd (k Pa)	Adratio
Boundary	Closely connected	62	1280.601 ± 2044.651	690.760 ± 843.965	30.560 ± 43.260	14.950 ± 23.245	3.072 ± 36.075	− 5.298 ± 26.048	0.516 ± 5.180	− 0.311 ± 3.900
	Hyperechoic halo	57	4478.101 ± 5671.559*	1642.758 ± 1925.874	291.792 ± 740.536*	24.607 ± 39.957	3.314 ± 670.758	− 0.184 ± 41.090	− 3.630 ± 16.922	1.808 ± 4.683*
Internal echo	Mixed echo	38	4006.894 ± 6037.150	1597.243 ± 2070.524	490.872 ± 1032.938	10.607 ± 20.471	− 13.572 ± 63.620	− 12.194 ± 39.163	− 9.183 ± 20.747	0.272 ± 3.340
	Low echo	81	2436.582 ± 3819.525	1012.039 ± 1307.210	53.683 ± 77.4456	22.334 ± 34.787	8.180 ± 490.083	− 0.248 ± 32.075	− 0.962 ± 7.324	0.797 ± 4.697
Posterior echo	No change	47	2243.592 ± 3927.642	898.829 ± 1527.459	258.631 ± 767.832	6.780 ± 13.110	− 3.809 ± 48.627	− 9.653 ± 29.280	− 5.587 ± 15.300	0.203 ± 2.673
	Enhance	35	1289.005 ± 2505.800	721.051 ± 937.549	21.374 ± 26.313	34.317 ± 40.921	4.350 ± 36.477	− 5.091 ± 26.986	− 1.341 ± 15.309	− 0.436 ± 5.979
	Attenuation	37	4950.947 ± 5693.742**	1876.636 ± 1722.928*	112.526 ± 118.505*	24.394 ± 36.910**	12.543 ± 70.792	8.939 ± 43.340	0.048 ± 10.846	2.443 ± 4.389
Blood flow	Not shown	21	5089.468 ± 4953.320	1533.134 ± 1792.920	69.486 ± 81.171	5.916 ± 6.257	3.063 ± 76.196	− 2.313 ± 41.786	− 1.413 ± 8.783	− 0.188 ± 2.593
	Intra-lesion signal	74	2868.813 ± 4536.686	1244.147 ± 1548.292	184.157 ± 584.223	23.621 ± 35.432	5.521 ± 54.161	− 2.093 ± 35.793	− 2.953 ± 13.353	1.081 ± 4.818
	Increased diffuse blood flow around the lesion	24	217.348 ± 315.284**	108.258 ± 143.059*	13.678 ± 13.409	4.152 ± 5.626	− 10.660 ± 10.735	− 8.840 ± 5.784	− 2.960 ± 2.337	− 1.100 ± 1.808
BI-RADS	4a	18	3390.180 ± 3873.428	1391.892 ± 1098.346	35.232 ± 34.624	8.940 ± 13.534	13.067 ± 62.156	1.850 ± 40.819	− 0.710 ± 8.141	1.233 ± 3.579
	4b	22	844.830 ± 1533.445	886.650 ± 969.893	39.128 ± 42.169	31.589 ± 45.310	− 17.475 ± 24.830	− 26.500 ± 14.517	− 5.138 ± 1.889	− 5.363 ± 4.455
	4c	79	2934.180 ± 4660.317	1136.973 ± 1607.474	133.575 ± 469.924	18.893 ± 31.593	4.789 ± 54.369	− 0.511 ± 33.973	− 2.706 ± 13.042	1.348 ± 3.905**

Afmax = [(cross-section maximum elasticity value) − (longitudinal-section maximum elasticity value)]^2^; Afmean = [(cross-section mean elasticity value) − (longitudinal-section mean elasticity value)]^2^; Afsd = [(cross-section stand deviation of elasticity value) − (longitudinal-section stand deviation of elasticity value)]^2^; Afratio = [(cross-section elasticity ratio) − (longitudinal-section elasticity ratio)]^2^; Admax = cross-section maximum elasticity value-longitudinal-section maximum elasticity value; Admean = cross-section mean elasticity value − longitudinal-section mean elasticity value; Adsd = cross-section stand deviation of elasticity value − longitudinal-section stand deviation of elasticity value; Adratio = cross-section elasticity ratio − longitudinal-section elasticity ratio; BI-RADS = breast imaging reporting and data system. Compared with corresponding subgroup, *P < 0.05, **P < 0.01

**Table 4:** Correlation between SWE anisotropy parameters and lesion measurement diameter (r)

Items	Emax (cross section)	Emax (longitudinal section)	Emean (cross section)	Emean (longitudinal section)	Esd (cross section)	Esd (longitudinal section)	Eratio (cross section)	Eratio (longitudinal section)
Distance between mass and skin	0.051	− 0.170	0.014	− 0.181	0.053	− 0.199	0.112	0.101
Maximum diameter of ultrasonic cross section	0.453**	0.182	0.501**	0.177	0.507**	0.152	0.360**	0.006
Maximum diameter of ultrasonic longitudinal section	0.169	0.341**	0.430*	0.372**	0.388**	0.296**	0.474**	0.155

Items	Admax	Admean	ADratio	Adsd	Afmax	Afmean	Afratio	Afsd
Distance between mass and skin	0.256*	0.236*	0.034	0.277*	0.142	0.064	0.252*	− 0.062
Maximum diameter of ultrasonic cross section	0.295**	0.359**	0.391**	0.214	0.272*	0.237*	0.121	0.068
Maximum diameter of ultrasonic longitudinal section	0.271*	0.301**	0.471**	0.104	0.304**	0.279*	0.117	0.135

*P < 0.05; **P < 0.01

### The SWE and anisotropy parameters of breast cancer are related to clinicopathological features

We further analyzed the difference between cross and longitudinal SWE anisotropy parameters in different clinicopathological characteristics of breast cancer. The results showed that the elastic modulus of breast cancer has no correlation with the patient's age, menopausal status, presence or absence of intraductal cancer, lymph node metastasis and different hormone receptors. However, breast cancers with different clinical stages or different histological grades had significant differences in elastic modulus. Moreover, the higher the clinical stage of breast cancer, the higher the cross-sectional Emax, Emean, and Esd. However, there were no statistically significant differences in Emax, Emean, and Esd of breast cancer in different clinical stages (Table 5). Therefore, breast cancer with higher clinical stage and histological grade also has higher elastic modulus.

**Table 5:** Differences in SWE parameters of cross and longitudinal section in different clinicopathological characteristics

Items	Grouping	E-maximum cross section diameter	N	E-maximum longitudinal section diameter	Emax (cross section)	Emax (longitudinal section)	Emean (cross section)	Emean (longitudinal section)	Esd (cross section)	Esd (longitudinal section)
Age (year)	≤ 46	19.577 ± 5.946	59	18.900 ± 0.954	102.180 ± 57.435	101.156 ± 62.885	68.338 ± 38.690	71.626 ± 40.393	16.362 ± 9.196	19.462 ± 15.969
	> 46	18.695 ± 6.269	60	17.965 ± 0.856	102.108 ± 62.454	96.738 ± 61.718	65.408 ± 42.831	68.068 ± 42.079	15.590 ± 9.098	18.168 ± 13.044
Menopausal state	Premenopause	19.664 ± 5.602	75	18.907 ± 5.804	103.324 ± 57.324	68.109 ± 38.616	68.584 ± 37.924	68.207 ± 36.522	16.125 ± 8.773	18.025 ± 13.873
	Post menopause	73.529 ± 50.564	44	17.325 ± 5.308	99.438 ± 65.864	62.892 ± 46.807	62.892 ± 46.807	73.529 ± 50.564	15.617 ± 9.985	20.596 ± 15.957
Clinical stage	≤ Stage 2	18.859 ± 5.564	64	18.391 ± 5.528	88.450 ± 52.468	95.564 ± 59.539	58.418 ± 35.578	67.420 ± 38.675	13.959 ± 7.991	18.530 ± 14.954
	> Stage 3	19.471 ± 6.759	55	18.471 ± 5.927	119.357 ± 64.277*	103.137 ± 65.455	77.460 ± 44.425*	72.846 ± 44.196	18.500 ± 9.860*	19.154 ± 14.075
Histological grade	Level 1	17.963 ± 4.995	27	16.700 ± 4.955	50.400 ± 17.907	56.763 ± 28.027	30.338 ± 13.930	40.775 ± 25.886	10.494 ± 1.756	12.281 ± 3.770
	Level 2	18.000 ± 5.664	45	17.689 ± 5.234	96.383 ± 51.845	108.626 ± 70.617	61.483 ± 31.936	74.477 ± 43.752	14.114 ± 7.571	21.143 ± 19.473
	Level 3	21.230 ± 6.902	47	20.407 ± 6.314	139.881 ± 61.615**	112.259 ± 55.712**	95.441 ± 42.337**	81.470 ± 38.276**	21.748 ± 10.643**	19.870 ± 9.432*
Intraductal carcinoma	No	19.390 ± 6.121	70	18.798 ± 5.693	101.435 ± 57.772	96.868 ± 61.691	66.998 ± 39.623	68.685 ± 39.943	15.585 ± 8.789	18.838 ± 15.903
	Have	18.864 ± 6.123	49	18.046 ± 5.698	102.869 ± 62.252	101.023 ± 62.922	66.708 ± 42.105	70.992 ± 42.605	16.367 ± 9.499	18.774 ± 13.074
Lymph nodes	Non-metastasis	17.159 ± 5.495	47	16.906 ± 5.452	96.618 ± 57.523	116.771 ± 81.259	62.459 ± 35.074	80.788 ± 50.560	14.435 ± 8.952	23.641 ± 22.570
	Metastasis	19.671 ± 6.173	72	18.844 ± 5.702	103.658 ± 60.580	94.024 ± 55.318	68.060 ± 42.174	66.818 ± 37.939	16.392 ± 9.161	17.481 ± 11.237
Hormone receptor status	ER positive group	18.725 ± 5.861	78	18.279 ± 5.425	101.821 ± 58.772	99.554 ± 61.429	65.410 ± 39.100	69.748 ± 39.773	15.694 ± 8.288	18.885 ± 14.704
	ER negative group	20.059 ± 6.344	41	18.900 ± 5.860	106.862 ± 61.826	100.000 ± 65.094	71.738 ± 43.631	70.910 ± 44.605	17.107 ± 10.334	19.245 ± 14.668

Items	Grouping	E-maximum cross section diameter	N	E-maximum longitudinal section diameter	Eratio (cross section)	Eratio (longitudinal section)	Paracancer E-maximum cross section diameter	Paracancer E-maximum longitudinal section diameter	Paracancer Emax (cross section)	Paracancer Emax (longitudinal section)
Age (year)	≤ 46	19.577 ± 5.946	59	18.900 ± 0.954	9.015 ± 6.049	8.803 ± 5.932	15.803 ± 6.706	14.226 ± 4.892	8.903 ± 3.840	8.790 ± 3.348
	> 46	18.695 ± 6.269	60	17.965 ± 0.856	8.535 ± 6.194	7.405 ± 4.391	15.980 ± 7.937	16.318 ± 9.269	9.515 ± 5.124	9.953 ± 6.774
Menopausal state	Premenopause	19.664 ± 5.602	75	18.907 ± 5.804	9.340 ± 5.798	8.285 ± 5.229	15.155 ± 6.118	14.556 ± 5.402	8.580 ± 3.756	8.736 ± 3.632
	Post menopause	73.529 ± 50.564	44	17.325 ± 5.308	7.471 ± 6.652	7.658 ± 5.294	17.583 ± 9.432	16.954 ± 10.784	10.663 ± 5.739	10.850 ± 7.954
Clinical stage	≤ Stage 2	18.859 ± 5.564	64	18.391 ± 5.528	8.732 ± 6.368	7.911 ± 5.665	14.430 ± 5.900	15.320 ± 5.357	8.261 ± 3.864	9.245 ± 3.695
	> Stage 3	19.471 ± 6.759	55	18.471 ± 5.927	8.823 ± 5.809	8.326 ± 4.679	17.731 ± 8.500*	15.240 ± 9.567	10.409 ± 5.028*	9.546 ± 6.973
Histological grade	Level 1	17.963 ± 4.995	27	16.700 ± 4.955	4.788 ± 3.183	6.125 ± 3.646	13.913 ± 4.946	11.031 ± 4.188	8.638 ± 4.428	6.619 ± 2.710
	Level 2	18.000 ± 5.664	45	17.689 ± 5.234	9.591 ± 7.328	8.706 ± 6.497	15.291 ± 6.758	16.157 ± 5.402	8.314 ± 3.742	9.700 ± 3.813
	Level 3	21.230 ± 6.902	47	20.407 ± 6.314	10.107 ± 4.751**	8.496 ± 3.986	17.811 ± 8.924	16.874 ± 10.149*	10.740 ± 5.279	10.659 ± 7.506**
Intraductal carcinoma	No	19.390 ± 6.121	70	18.798 ± 5.693	9.070 ± 6.180	8.518 ± 5.435	14.590 ± 7.160	15.330 ± 6.205	8.945 ± 4.179	8.775 ± 4.916
	Have	18.864 ± 6.123	49	18.046 ± 5.698	8.467 ± 6.058	7.662 ± 5.029	15.997 ± 7.793	16.469 ± 8.333	9.487 ± 4.879	9.997 ± 5.781
Lymph nodes	Non-metastasis	17.159 ± 5.495	47	16.906 ± 5.452	10.865 ± 8.113	9.306 ± 7.426	12.724 ± 5.414	15.729 ± 4.995	7.176 ± 3.647	10.159 ± 3.744
	Metastasis	19.671 ± 6.173	72	18.844 ± 5.702	8.198 ± 5.346	7.763 ± 4.460	16.761 ± 7.553	15.163 ± 8.040	9.771 ± 4.597*	9.165 ± 5.733
Hormone receptor status	ER positive group	18.725 ± 5.861	78	18.279 ± 5.425	8.867 ± 6.267	8.219 ± 5.222	15.585 ± 6.822	15.110 ± 6.156	9.265 ± 4.445	8.865 ± 4.332
	ER negative group	20.059 ± 6.344	41	18.900 ± 5.860	8.962 ± 5.954	8.228 ± 5.335	16.086 ± 7.986	14.941 ± 9.110	9.217 ± 4.794	9.734 ± 6.524

Emax = maximum elasticity; Emean = mean elasticity; Esd = stand deviation; Eratio = elasticity ratio. Compared with corresponding subgroup, *P < 0.05; **P < 0.01

**Fig. 2:**
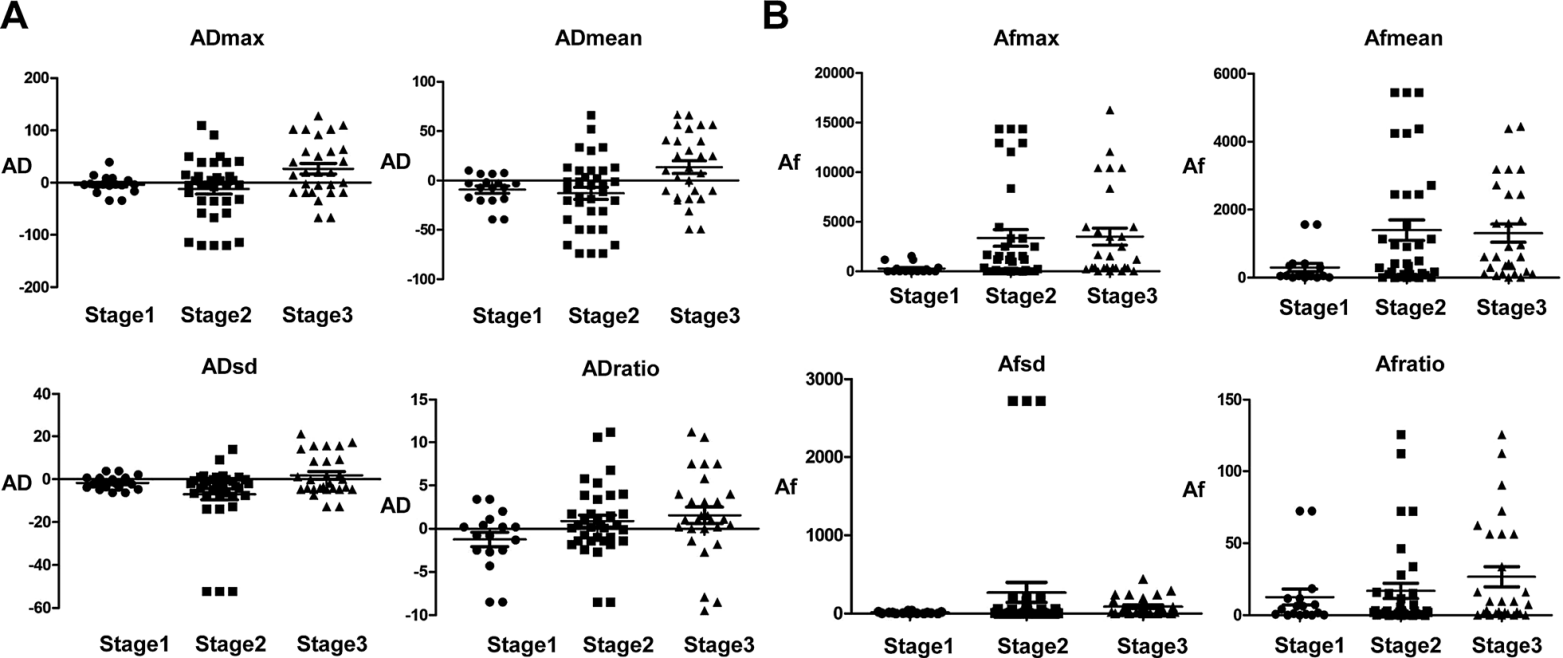
Differences in SWE anisotropy indexes of breast cancer with different histological grades. **A** The difference of SWE anisotropy in different histological grades. **B** The difference of SWE anisotropy factor in different histological grades

Moreover, the anisotropy parameters of SWE of breast cancer were significantly related to histological grade (Fig. 2A and B), indicating that breast cancer with higher histological grade has obvious SWE anisotropy.

### Immunohistochemistry and real-time PCR analysis of CSCs and EMT markers in breast cancer

**Fig. 3:**
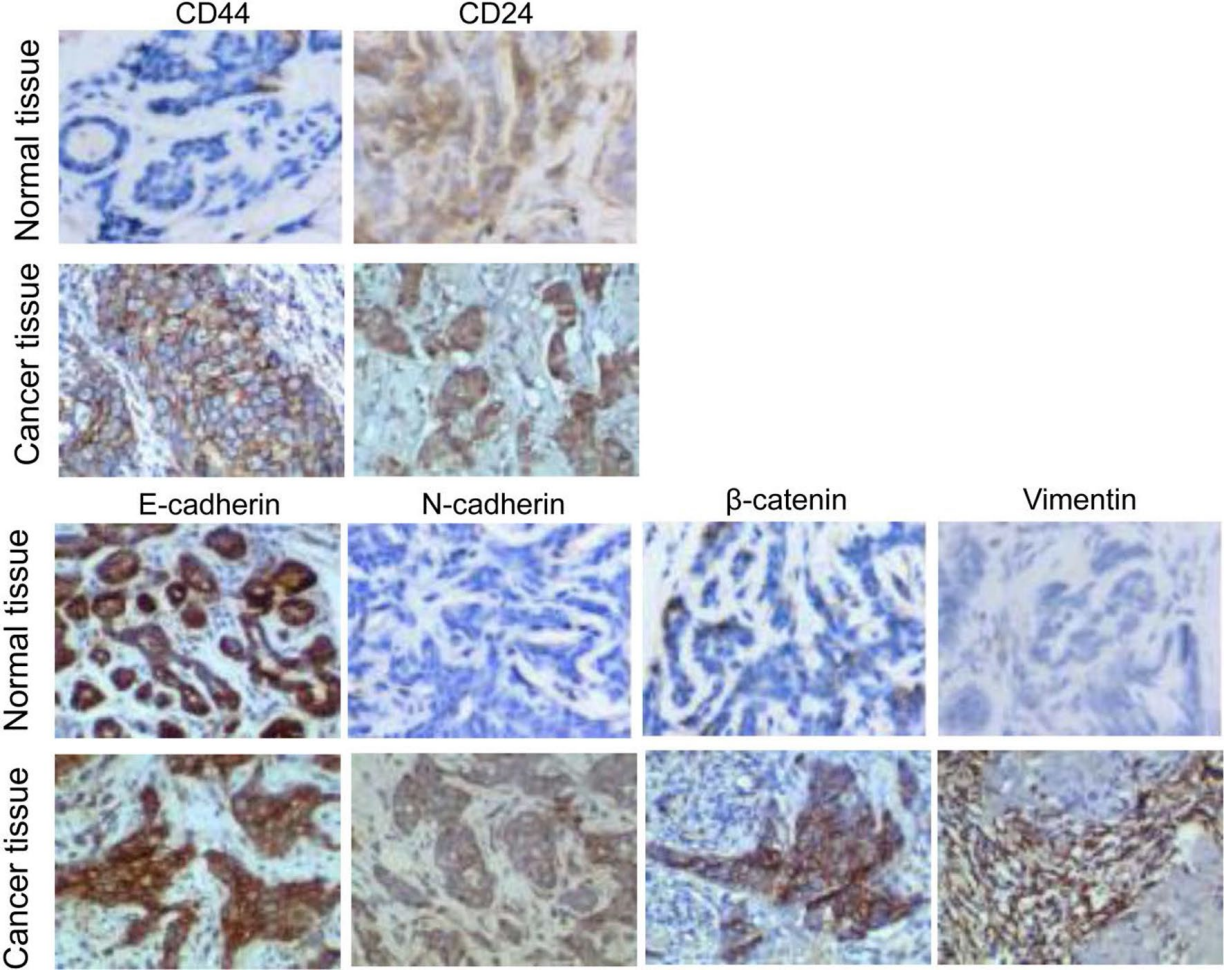
Representative immunohistochemical staining results. Magnification: × 200. CD44 was strongly positively expressed in the cell membrane and cytoplasm of cancer cells (+++). CD24 was weakly positively expressed in the cytoplasm of cancer cells (+). E-cadherin was weakly positively expressed in the cell membrane and cytoplasm of cancer cells (+). N-cadherin was strongly positively expressed in the cell membrane of cancer cells (+++). β-catenin was positively expressed in the cell membrane of cancer cells (++). Vimentin was strongly positively expressed in the cytoplasm of the tissue surrounding the cancer nest (+++)

**Fig. 4:**
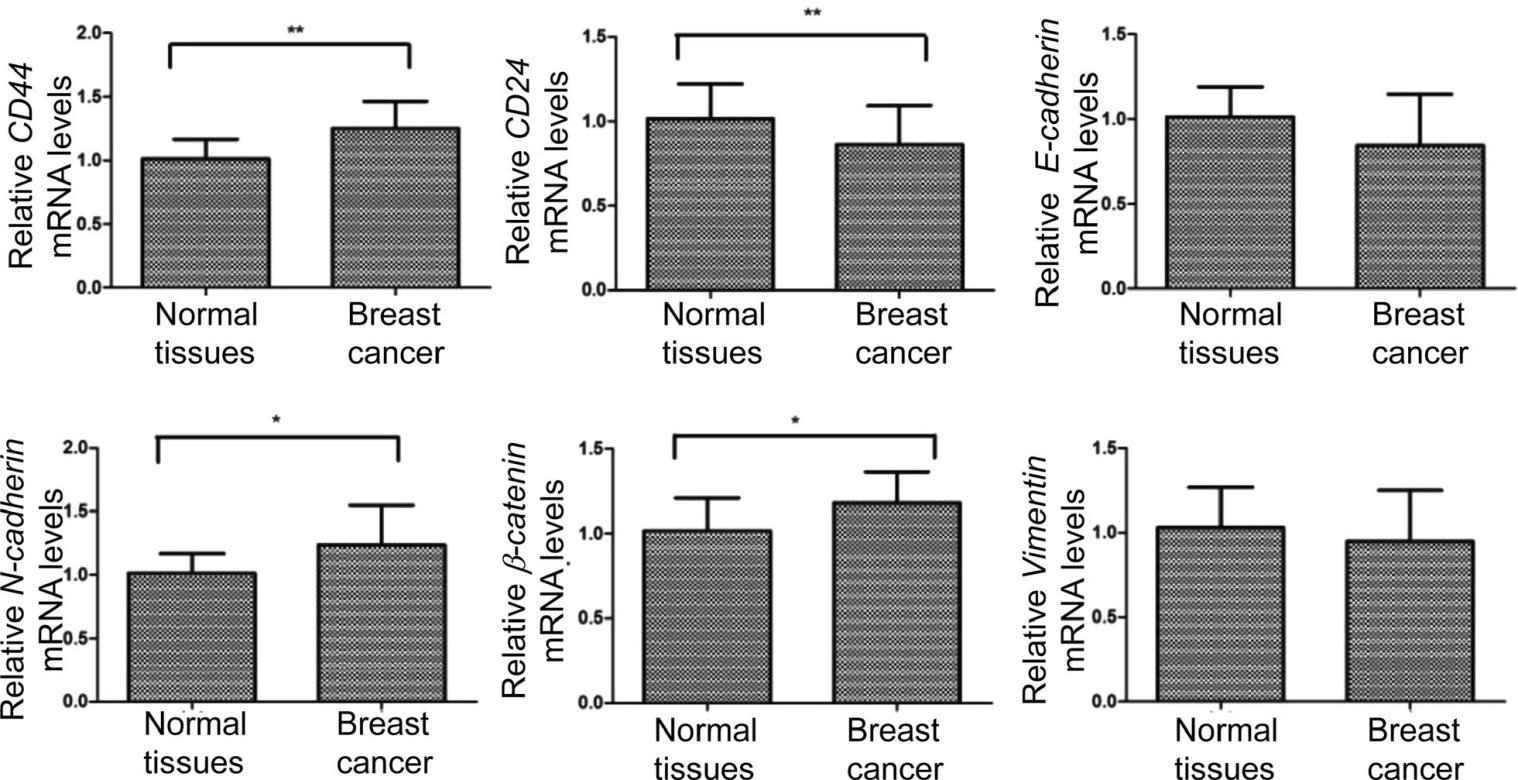
The mRNA levels of CSCs and EMT related genes. Real-time PCR was used to detect mRNA levels of each CSCs and EMT marker gene. *P < 0.05; **P < 0.01

Taking adjacent tissues as a control, immunohistochemical results showed that breast cancer tissues had significantly high expression of CD44 and significantly low expression of CD24 (Fig. 3 and Table 6). In addition, the expression of N-cadherin, β-catenin, and vimentin was high (*p* < 0.05). However, the expression of E-cadherin in cancer and adjacent tissues was not statistically significant (*p* > 0.05, Table 6). Real-time PCR showed that *CD44, N-cadherin,* and *β-catenin* mRNAs were highly expressed in cancer tissues, while *CD24* mRNAs were low in expression compared with adjacent tissues (*P* < 0.05) (Fig. 4 and Table 7). There was no statistically significant difference in the expression of *Vimentin* and *E-cadherin* mRNAs between cancer and adjacent tissues (Fig. 4 and Table 7). Both the immunohistochemical results and real-time PCR results showed that breast cancer tissues showed the characteristics of CSCs and EMT.

**Table 6:** Immunohistochemical scores of CSCs and EMT in breast cancer tissues (n = 119)

Groups	CD24	CD44	E-cadherin	N-cadherin	β-catenin	Vimentin
Cancer tissue	1.333 ± 1.047	2.931 ± 1.241	2.600 ± 1.242	2.017 ± 1.235	2.586 ± 1.200	3.431 ± 0.797
Paracancer tissue	2.259 ± 1.278	1.533 ± 1.060	2.948 ± 1.343	0.200 ± 0.561	1.533 ± 1.125	1.933 ± 0.961
*Z*	− 2.531	− 3.828	− 1.284	− 4.634	− 2.984	− 4.681
*P*	0.011	< 0.001	0.199	< 0.001	0.003	< 0.001

**Table 7:** The relative mRNA level of each gene (n = 63)

Groups	*E-cadherin*	*β-catenin*	*Vimentin*	*N-cadherin*	*CD44*	*CD24*
Normal	1.013 ± 0.179	1.015 ± 0.194	1.009 ± 0.143	1.010 ± 0.155	1.011 ± 0.155	1.017 ± 0.205
Breast cancer	0.844 ± 0.304	1.178 ± 0.184^Δ^	0.948 ± 0.300	1.237 ± 0.308^Δ^	1.250 ± 0.216^Δ^	0.861 ± 032.2^Δ^

Compared with normal, ΔP < 0.05

### The SWE and anisotropy are related to CSCs and EMT

In order to further clarify the prognostic value of SWE and anisotropy, we conducted correlation analysis on SWE modulus, anisotropy, and markers of CSCs and EMT (Table 8). The results showed that mRNA level of *E-cadherin* was negatively correlated with Emax, Emean, Esd, and Eratio on the longitudinal section, negatively correlated with Eratio on the cross section, and negatively correlated with Afmean. These results indicated that the higher the stiffness of the longitudinal section, the lower expression of E-cadherin in epithelial cells. In addition, *β-catenin* mRNA was positively correlated with Emax, Emean, and Esd of the cross section, and positively correlated with Admean and Adsd. These results indicated that the higher the stiffness of the cross section, the higher the expression of the epithelial phenotype β-catenin. *N-cadherin* mRNA was positively correlated with Emax, Emean, and Esd of the longitudinal and cross sections, and was also positively correlated with the maximum diameter of the lesions on the cross and longitudinal sections, indicating that the increases of stiffness of the longitudinal section and the cross section, and the lesion enlargement were always accompanied by the increase of N-cadherin. In addition, Admean and *N-cadherin* mRNA were positively correlated, indicating that the greater difference in stiffness between the longitudinal section and the cross section, the more obvious the interstitial phenotype. *CD44* mRNA was positively correlated with Emax, Emean, Esd, and Eratio of longitudinal and cross sections, and was positively correlated with all anisotropy factors of Afmax, Afmean, Afratio, and Afsd, indicating that the greater the difference in stiffness between the longitudinal section and the cross section, the more obvious the phenotype of CSCs. However, the expression of *Vimentin* and *CD24* mRNAs had no correlation with SWE parameters.

**Table 8:** Correlation of SWE elastic modulus and anisotropy parameters with tumor stem cell and epithelial-mesenchymal transition markers

Items	E-maximum cross section diameter	E-maximum longitudinal section diameter	Emax (cross section)	Emax (longitudinal section)	Emean (cross section)	Emean (longitudinal section)	Esd (cross section)	Esd (longitudinal section)	Eratio (cross section)	Eratio (longitudinal section)
E-cadherin	− 0.049	− 0.089	− 0.193	− 0.285*	− 0.212	− 0.300**	− 0.186	− 0.247*	− 0.299**	− 0.232*
β-catenin	0.2	0.141	0.314**	0.008	0.283*	0.000	0.288*	− 0.065	− 0.003	− 0.127
Vimentin	0.091	0.13	− 0.148	− 0.057	− 0.179	− 0.057	− 0.147	− 0.07	− 0.221	− 0.164
N-cadherin	0.306**	0.257*	0.600**	0.415**	0.617**	0.419**	0.610**	0.257*	0.087	0.054
CD44	0.149	0.146	0.646**	0.560**	0.606**	0.532**	0.641**	0.509**	0.576**	0.553**
CD24	0.016	− 0.039	− 0.063	− 0.178	− 0.077	− 0.172	− 0.117	− 0.125	0.04	− 0.052

Items	Admax	Admean	ADratio	Adsd	Afmax	Afmean	Afratio	Afsd
E-cadherin	0.116	0.108	− 0.138	0.154	− 0.179	− 0.223*	0.055	− 0.182
β-catenin	0.343**	0.338**	0.145	0.293**	0.001	− 0.043	0.016	− 0.132
Vimentin	− 0.1	− 0.145	− 0.11	− 0.028	− 0.122	− 0.108	− 0.133	− 0.097
N-cadherin	0.189	0.232*	0.055	0.152	0.095	0.043	0.075	− 0.036
CD44	0.072	0.082	0.141	− 0.129	0.642**	0.602**	0.253*	0.467**
CD24	0.137	0.116	0.117	0.061	0.061	0.047	0.077	− 0.006

*P < 0.05; **P < 0.01

## Discussion

### Anisotropy of SWE

Anisotropy is a term describing directional dependence, which exists in fiber-rich biological tissues. Previous study has confirmed the anisotropy of shear waves in skeletal muscle, myocardium, tendons, and other tissues [15]. Recent study has suggested that the elastic characteristics of normal breast and adipose tissue were also anisotropic [16]. In this study, SWE was used to confirm the anisotropy of breast cancer. The cross section elastic quantitative parameters of breast cancer, such as Emax, Emean, and Esd, were significantly higher than the longitudinal section, suggesting that the stiffness of breast lesions is anisotropic in two perpendicular sections, and the elastic modulus of the cross section is more sensitive than that of the longitudinal section. This may be caused by the shrinkage of tumor cells and the arrangement of collagen fibers at the boundary of the lesion. The tumor invades along the direction of the collagen fiber structure, and then metastasizes, and the largest diameter of the tumor is formed [17], which indirectly explains why the shear wave may spread faster in the growth direction of the tumor. Tumor stiffness is related to the connective tissue proliferative response around the tumor, stromal edema around the tumor, internal tumor fibrosis, calcification, and necrosis [18, 19]. The increase in SWE parameters is caused by compression and reduction of the extracellular space, which is caused by the proliferation of tumor cancer cells and tumor stromal cells [20]. The increase in SWE parameters is also derived from the abnormality of the breast cancer nuclei, that is, the shrinkage of the intracellular space caused by the increase and enlargement of the tumor cell nuclei [21]. The stiffness of the tumor increases, and the SWE elastic modulus value of breast cancer also increases, which may lead to more obvious the difference in stiffness in mutually perpendicular sections. This may because the speed of the pulse wave emitted by SWE in the direction perpendicular to the tumor stroma is obviously different from the direction parallel to the tumor stroma.

### SWE anisotropy is reflected in its correlation with conventional ultrasound

In this study, the SWE parameters of breast cancer with better correlation with conventional ultrasound were cross section Emax, longitudinal section Emax, and cross section Emean. The SWE anisotropy parameters of breast cancer that had a good correlation with conventional ultrasound included Admax, Admean, and Adsd. These results indicate that the SWE value of the cross-section has a higher correlation with the ultrasound characteristics, and Emax has the highest diagnostic efficiency among the indicator of SWE. Studies [9, 22] have shown that breast cancer with malignant features on conventional ultrasound usually has a higher stiffness in the cross section, and the stiffness difference between the longitudinal section and the cross section is also greater. This is because the characteristics of the invasive growth of breast cancer, which lead to increased necrosis and repair around the lesion, and result in increased fibrous components, adhesions, and the formation of the surrounding interstitial infiltration zone [23]. The interstitial infiltration zone corresponds to the hyperechoic halo in conventional ultrasound. The dense arrangement of fibrous components in the breast cancer can cause the posterior echo attenuation, and liquefaction and necrosis in the breast cancer can form a mixed echo. These all lead to a decrease in elasticity and an increase in stiffness of the aggressive breast cancer [23]. This study showed that there was no statistical correlation between blood flow and elastic modulus values. However, the blood flow was correlated with anisotropic factors, suggesting that the amount of blood supply does not affect the stiffness of breast cancer, but the SWE anisotropy of breast cancer with poor blood supply is more obvious. This also suggests that a large number of abnormally functioning, disorderly arranged new blood vessels may affect the compactness of the structure of the tumor stroma, thereby affecting the anisotropy [24]. Most of the parameters of SWE in this study had good correlation with BI-RADS, which also confirms that SWE may have a good diagnostic value for breast lesions. However, there was no statistical correlation between the anisotropy parameter and BI-RADS, which indicates that its value in breast cancer diagnosis may be not as good as the elastic modulus value. The correlation between the anisotropy parameters of SWE and the measured diameter of the lesions indicates that the anisotropy parameters are more likely to be affected by the size and depth of the mass than the Young's modulus value. Studies [25, 26] have shown that the larger the lesion, the harder the breast cancer, and the more obvious the anisotropy. However, the larger the diameter of the longitudinal section, the higher the stiffness of the cross section. This is because larger breast cancers often have obvious peripheral connective tissue proliferative responses, interstitial edema around the tumor, internal tumor fibrosis, calcification, and necrosis, making the heterogeneity of the tumor more obvious [27]. The tumor grows along the direction of the collagen fiber structure, that is, the longitudinal section, and the tumor cells on the cross section face the barrier of the collagen fiber structure, which causes the compression and reduction of the extracellular space to form a higher stiffness.

### The anisotropy of SWE is reflected in its correlation with clinicopathological manifestations

SWE modulus and anisotropy are determined by breast cancer tumor stroma, and the formation and development of breast cancer tumor stroma affects the morphology, proliferation, migration, invasion and EMT of tumor cells [28]. In this study, we confirmed that the SWE parameters and anisotropy parameters of the cross and longitudinal sections were correlated with some clinicopathological manifestations. When the histological grade is higher, breast cancer has greater stiffness and more obvious SWE anisotropy. The higher the histological grade, the higher the rate of DNA aneuploidy in the nucleus reflecting the growth and differentiation of tumor cells. The tumor’s ability to infiltrate the surrounding structures and the range of infiltration also increase; the proliferative activity and cell density of tumor cells also increase; the tumor cell nucleus and nucleolus also increase; the stiffness of the tissue increases; and, the interstitial fiber framework of the tumor also tends to be perfect, all of which may lead to more obvious anisotropy in SWE [29]. Breast cancer with a higher clinical stage has a higher modulus of elasticity in the cross section, but there is no such correlation in the longitudinal section, which also indicates that the tumor stiffness in the cross section can better reflect the prognosis than the longitudinal section [30, 31]. In this study, there was no difference in SWE between patients with or without ductal carcinoma in situ. The main reason might be due to the complexity of ductal carcinoma in situ. One study has shown that the average Young's modulus value of ductal carcinoma in situ of medium and high nuclear grade is higher than that of ductal carcinoma in situ of low nuclear grade, which is lower than that of normal glands [32].

### SWE anisotropy is related to the markers of CSCs and EMT

Here, we showed that the E-cadherin was negatively correlated with the longitudinal section stiffness, while β-catenin was positively correlated with the cross section stiffness. These results also confirmed that the anisotropy of stiffness was related with the expression of epithelial phenotype makers. The N-cadherin and CD44 had correlations with most SWE parameters, and also showed the best correlation with the anisotropy of different cross-sectional stiffness, indicating that the greater the stiffness of breast cancer, the greater the difference in the stiffness of the cross section. The high expression of CSCs and EMT markers also indicate the worse prognosis of breast cancer. Some study believed that the change in matrix stiffness can trigger the collective migration of cells by promoting the transformation of epithelial cells to mesenchymal cells [33], which also confirms that the increase in tumor stiffness can strengthen the EMT and anisotropy of breast cancer and other tumors. EMT allows differentiated breast epithelial cells to acquire the characteristics of CSCs and breast CSCs often differentiate into cells with different heterogeneities in many ways, and the structure of tumor mesenchyme is also more complicated [34], which makes the heterogeneity of tumor structure more obvious. Different areas and different planes may have different tumor stiffness. The most intuitive imaging manifestation of this stiffness heterogeneity is that there are different Young's moduli of SWE in different areas. The quantitative parameters of SWE can reflect the tumor heterogeneity of breast cancer stiffness at the same level, and the anisotropy parameters of SWE can also reflect the tumor heterogeneity of breast cancer at different levels. The stiffness and anisotropy of breast cancer tumors can be directly displayed through the macroscopic images of SWE, which indirectly predicts the CSCs and EMT of breast cancer.

## Conclusions

In conclusion, this study believes that each parameter of SWE has prognostic value. The cross-sectional SWE parameter is better than the longitudinal SWE parameter. Emax is better than Emean. The anisotropy parameter of SWE is better than the SWE parameter, and the anisotropy factor is better than the anisotropy difference. Our research is helpful to guide the clinical assessment of breast cancer prognosis through non-invasive and convenient imaging.

## Data Availability

The raw data used and/or analysed during the current study are available from the corresponding author on reasonable request. Dataset deposition is not applicable to this article as no datasets were generated or analysed during the current study.

## Abbreviations

SWE: Shear wave elastography
CSCs: Cancer stem cells
EMT: Epithelial-mesenchymal transition
Emax: Maximum elasticity value
Emean: Average elasticity value
Esd: Standard deviation of the lesion elasticity value
Eratio: Elasticity value of adipose tissue
AD: Anisotropic difference
AF: Anisotropy factor
BI-RADS: Breast imaging reporting and data system
